# Adipokine patterns in type 1 and type 2 diabetes mellitus: comparative analysis of adiponectin–leptin ratio, visfatin, and TNF-α

**DOI:** 10.3389/fendo.2026.1837854

**Published:** 2026-07-22

**Authors:** Bo Wu, Huixiang He, Jinlong Zeng, Xiaoli Lai, Yan Bai

**Affiliations:** 1 Department of Transfusion, The Fourth Affiliated Hospital, Guangzhou Medical University, Guangzhou, Guangdong, China; 2 Department of Clinical Laboratory, The Fourth Affiliated Hospital, Guangzhou Medical University, Guangzhou, Guangdong, China; 3 Department of Haematology and Rheumatology, The Fourth Affiliated Hospital, Guangzhou Medical University, Guangzhou, Guangdong, China; 4 Department of Infectious Diseases, The Fourth Affiliated Hospital, Guangzhou Medical University, Guangzhou, Guangdong, China; 5 Department of Pathology, The Fourth Affiliated Hospital, Guangzhou Medical University, Guangzhou, Guangdong, China

**Keywords:** adipokines, adiponectin–leptin ratio, TNF-α, type 1 diabetes mellitus, type 2 diabetes mellitus, visfatin

## Abstract

Adipokines play an important role in metabolism, insulin sensitivity, and inflammation, and may provide distinct contributions to the pathophysiology of type 1 diabetes mellitus (T1DM) and type 2 diabetes mellitus (T2DM). The differences in roles of the adiponectin-leptin ratio (ALR), visfatin, and tumor necrosis factor-alpha (TNF-α) in the various subtypes of diabetes are still not fully understood. This cross-sectional study involved 141 participants, divided into healthy controls (n = 47), patients with T1DM (n = 47), and patients with T2DM (n = 47). Serum adiponectin, leptin, visfatin, TNF-α, fasting plasma glucose (FPG), glycated hemoglobin (HbA1c), and serum insulin levels were measured. The ALR was calculated. Insulin resistance and β-cell function were examined using homeostasis model assessment (HOMA) indices. We performed group comparisons, correlation studies, logistic regression tests, and receiver operating characteristic (ROC) curve analyses. The differences observed were all statistically significant. Participants with T2DM showed a more adverse metabolic profile, characterized by greater waist circumference and higher levels of FPG, HbA1c, serum insulin, leptin, and TNF-α, along with lower levels of adiponectin and ALR. Participants with T1DM also exhibited altered adipokine profiles compared to controls, notably with increased levels of leptin and TNF-α. All diabetic groups had decreased levels of visfatin, with the T2DM group showing the largest reduction. ROC analysis demonstrated effective discrimination of TNF-α, ALR, and visfatin in this group. This study highlights the different inflammatory and adipokine profiles across diabetes subtypes. The assessment of these biomarkers enhances the understanding of metabolic differences between T1DM and T2DM. However, these findings are exploratory and require validation in large, controlled studies.

## Introduction

Diabetes mellitus is a chronic metabolic disorder characterized by hyperglycemia resulting from impaired insulin secretion, insulin deficiency, insulin resistance, or a combination of these mechanisms (1). Type 1 diabetes mellitus (T1DM) and Type 2 diabetes mellitus (T2DM) are the two major forms of diabetes, and they markedly differ in pathophysiology (2). While T1DM is characterized by the autoimmune destruction of pancreatic beta cells, leading to an absolute deficiency of insulin, T2DM is characterized by insulin resistance, progressively dysfunctional beta cells, overweight, and chronic low-grade inflammation (3). Though both types of diabetes result in the impaired regulation of glucose, the metabolic and inflammatory profiles for T1DM and T2DM are distinct (4).

In the past few years, the role of adipose tissue in diabetes has changed. Instead of solely being seen as an energy storage tissue, it has now been recognized as an active endocrine organ, which is capable of secreting a host of bioactive molecules capable of modulating glucose metabolism, insulin sensitivity, lipid balance, and inflammation (5). Two of the major adipokines involved in metabolic disorders are adiponectin and leptin. While adiponectin has insulin-sensitizing and anti-inflammatory effects, leptin has a role in the control of appetite, the regulation of energy balance, and the promotion of inflammation. For these reasons, the adiponectin to leptin ratio (ALR) is an emerging marker of dysfunctional adipose tissue and increasing metabolic risk (6).

Other inflammatory mediators and adipokines may further refine the metabolic description of diabetes. Visfatin has been associated with both inflammatory pathways and the processes of insulin secretion and glucose metabolism, but its precise role in diabetes remains incompletely defined. In particular, it is uncertain whether circulating visfatin is compensatory or pathogenic, whether its direction of change varies with disease stage, adiposity, assay method, and treatment status, whether it differs between diabetes subtypes, and whether it primarily reflects beta-cell function, inflammation, or adipose tissue dysfunction. In chronic metabolic inflammation and the development of insulin resistance, a crucial role is played by the pro-inflammatory cytokine tumor necrosis factor-alpha (TNF-α) (7). TNF-α has been measured at higher levels in both T1DM and T2DM, although different diabetes types may show different magnitudes and patterns of these changes (8).

Adipokines have stimulated interest, and although a substantial body of literature has been produced, the majority has focused on individual markers rather than considering the pattern of multiple adipokines integrated across the types of diabetes (9). In Asian populations, and particularly in China, there is a paucity of data comparing ALR, visfatin, and TNF-α among healthy individuals and patients with T1DM and T2DM. This gap is clinically important because the prevalence of diabetes has risen rapidly in China and across Asia and now represents a major regional disease burden (10, 11). East Asian populations, including Chinese populations, also show a metabolic phenotype that differs from that of European populations. In these populations, T2DM tends to develop at lower body mass index (BMI) thresholds and at a younger age, with a predisposition to central and visceral adiposity, early insulin resistance, and relatively prominent beta-cell dysfunction (10, 12). These features, together with the regional burden of disease, make region-specific evaluation of adipose-derived and inflammatory biomarkers in diabetes particularly informative. Understanding these relationships may facilitate the refinement of metabolic phenotyping and the identification of important biomarker patterns associated with various types of diabetes (9).

Considering these markers together, rather than in isolation, may provide a more integrated picture of the metabolic and inflammatory disturbances in diabetes than single-biomarker studies. The ALR reflects the balance between insulin-sensitizing, anti-inflammatory adiponectin and pro-inflammatory leptin, and therefore serves as an indicator of adipose tissue dysfunction and reduced insulin sensitivity. TNF-α represents inflammatory signaling that interferes with insulin receptor signaling and promotes insulin resistance. Visfatin may operate at the interface of adipose tissue, inflammation, beta-cell function, and glucose metabolism. Mechanistically, these pathways converge on the core features of diabetes. Adipose tissue dysfunction increases the release of pro-inflammatory adipokines and free fatty acids, which sustain chronic low-grade inflammation and activate inflammatory signaling cascades such as those mediated by TNF-α. This in turn impairs insulin signaling and contributes to insulin resistance, while persistent metabolic and inflammatory stress accelerates beta-cell dysfunction. Evaluating ALR, visfatin, and TNF-α simultaneously across T1DM and T2DM may therefore capture this combined adipose, inflammatory, and glycemic axis more completely than assessment of any single marker alone.

Therefore, this study evaluated serum visfatin, TNF-α, and the ALR among healthy controls and patients with T1DM and T2DM. Moreover, we analyzed the relationships of these variables with the anthropometric and glycemic parameters in order to comprehensively define the metabolic and inflammatory disruptions caused by diabetes mellitus.

## Materials and methods

This was a cross-sectional, single-center study conducted at the Fourth Affiliated Hospital of Guangzhou Medical University. Participants were recruited consecutively during the study period. The study design was approved by the Ethics Committee of the Fourth Affiliated Hospital of Guangzhou Medical University (Approval No. 2022-H-036). The study was conducted in full adherence to the Declaration of Helsinki. Written informed consent was obtained for all study participants. The legal guardians gave informed consent for the minors. A total of 141 participants were enrolled in the study and were classified into three groups comprising healthy controls (n = 47), T1DM (n = 47), and T2DM (n = 47). The study participants were obtained using the consecutive sampling method at the Fourth Affiliated Hospital of Guangzhou Medical University. Using this consecutive sampling approach, potential participants were enrolled in the study as they presented at the study site, during the study recruitment period, until the target sample size for the study was met for each group. The method constituted non-random, consecutive sample recruitment.

The age range of the study population was 5 to 70 years for both sexes. Participants with T1DM and T2DM were included based on American Diabetes Association (ADA) criteria. Patients with T2DM were eligible only if newly diagnosed or diagnosed within the preceding year, that is, with a disease duration of less than one year. Information on disease duration in T1DM participants was recorded where available; however, unlike T2DM, T1DM duration was not restricted to newly diagnosed cases. Healthy controls were classified as individuals with no history of diabetes and major chronic illnesses and had fasting plasma glucose (FPG) levels of less than 100 mg/dL. Patients with chronic diseases of the liver, kidneys, and heart, autoimmune diseases, inflammatory diseases, acute infections, pregnancy or gestational diabetes, and any other endocrine disorders were excluded from the study. Information regarding disease duration and ongoing treatment was reviewed, where available. Medication use, including insulin and oral antidiabetic therapy, was not uniformly controlled and may have influenced adipokine and inflammatory marker levels.

Demographic variables, including age, sex, and BMI, together with clinical variables including height and weight, were collected using a systematic format. Blood pressure (BP) was recorded as systolic blood pressure (SBP) and diastolic blood pressure (DBP). For anthropometric evaluation, waist circumference (WC), hip circumference (HC), and waist-to-hip ratio (WHR) were also measured and analyzed in accordance with standard operating procedures (SOPs). Blood samples for biochemical analysis were taken after an overnight fast of 10–12 hours. FPG was measured after the overnight fast, and the HbA1c level was assessed by the method of high-performance liquid chromatography (HPLC). Evaluation of serum insulin level was done by an immunoassay kit (DiaMetra, Italy). Enzyme-linked immunosorbent assay (ELISA) kits (Bioassay Technology Laboratory, United Kingdom) were used to quantify circulating levels of adiponectin, leptin, visfatin, and TNF-α. The absorbance was measured using a microplate reader (DR-200Bs, Diatek, United States) in accordance with the manufacturer’s protocol. Serum samples were processed uniformly prior to analysis, and all assays were conducted according to the manufacturer’s protocol.

The ALR was obtained from the ratio of adiponectin to leptin. The Homeostasis Model Assessment (HOMA) indices were used to assess insulin resistance and beta (β) cell function. Homeostasis model assessment of insulin resistance (HOMA-IR) was determined as the product of fasting insulin and fasting glucose divided by 22.5. Homeostasis model assessment of beta-cell function (HOMA-B) was calculated as 20 × fasting insulin/(fasting glucose − 3.5), with fasting glucose expressed in mmol/L.

No formal *a priori* power calculation was performed. The sample size of 47 participants per group was determined based on the availability of eligible participants during the recruitment period and the exploratory nature of this single-center biomarker study, and is comparable to that of previous biomarker studies in diabetes. Therefore, the findings should be interpreted as hypothesis-generating and require confirmation in larger, adequately powered cohorts.

Participants in this study were both pediatric and adult, aged 5 to 70 years. This heterogeneity likely resulted in different metabolic and inflammatory profiles for each age group. Age was considered a possible confounding variable, and an imbalance across the study groups was attributed to age. Future studies should ideally aim for both age-matched recruitment and pediatric/adult subgroup analyses. Age-adjusted multivariable analyses could also be implemented to identify pediatric and adult diabetes-related biomarker discrepancies. These analyses should be considered when interpreting the present study’s results.

Statistical analysis was undertaken with IBM SPSS Statistics (version 26.0) and the R software program for the purposes of data visualization and plotting of receiver operating characteristic (ROC) curves. Continuous variables are presented as mean ± standard deviation (SD). The normality of data distribution was tested using the Shapiro-Wilk test. For normally distributed variables, one-way analysis of variance (ANOVA) was used, and for non-normally distributed variables, the Kruskal–Wallis test, with appropriate *post hoc* analyses (which could be determined from the distribution of the data and design of the study). The significance level was set at p < 0.05. As noted above, the observed differences in age and adiposity between groups were considered potential confounders when interpreting the results.

Categorical variables were analyzed using the Chi-square test. For all biochemical and clinical parameters, Pearson correlation analysis was used. To determine the association of adipokines and metabolic variables with diabetes status, multinomial logistic regression analysis was used, and results were expressed as odds ratios (OR) with 95% confidence intervals (CI). The diagnostic evaluation of the selected biomarkers (as described) involved ROC curve analysis, with data visualization and ROC curve plotting undertaken in R using ggplot2, ggpubr, and pROC packages. The significance level was set at p < 0.05.

## Results

The final analysis included 141 participants, with 47 individuals in each group. The groups differed significantly in age, with the T1DM group being younger and the T2DM group being older than controls (**Table 1**, p < 0.05), reflecting an age imbalance across the study groups. **Table 1** summarizes the baseline data and the anthropometric information regarding the study population. Compared with the control and T1DM groups, participants with T2DM showed significantly higher WC and WHR values (p < 0.05), indicating greater central adiposity. No significant differences in SBP or DBP were observed across the groups (**Table 1**, p > 0.05).

**Table 1:** Baseline and anthropometric characteristics.

Variable	Control (n=47)	T1DM (n=47)	T2DM (n=47)	p-value
Age (years)	30.36 ± 1.11	14.46 ± 0.71	42.13 ± 0.74	<0.05
BMI (kg/m²)	27.65 ± 1.04	21.47 ± 1.17	30.94 ± 0.87	<0.05
WC (cm)	92.55 ± 0.99	100.06 ± 1.14	101.97 ± 1.46	<0.001
HC (cm)	98.74 ± 3.18	93.38 ± 3.32	89.68 ± 1.66	<0.001
WHR	0.93 ± 0.05	1.07 ± 0.04	1.13 ± 0.04	<0.001
SBP (mmHg)	114.65 ± 3.07	124.97 ± 3.19	134.97 ± 3.09	>0.05
DBP (mmHg)	82.34 ± 1.68	92.36 ± 2.03	98.36 ± 1.48	>0.05

Metabolic and glycemic parameters are summarized in **Table 2**. FPG and HbA1c levels were significantly elevated in both diabetic groups compared with controls, with the highest values observed in T2DM (**Table 2**, p < 0.001). Serum insulin levels were lowest in T1DM and highest in T2DM (**Table 2**, p < 0.001). HOMA-IR values were increased in T2DM, while HOMA-B was reduced in T1DM (**Table 2**, p < 0.05).

**Table 2:** Metabolic and glycemic parameters.

Variable	Control	T1DM	T2DM	p-value
Serum Insulin (µIU/mL)	14.70 ± 1.78	8.64 ± 1.13	22.10 ± 1.46	<0.001
FPG (mmol/L)	4.89 ± 0.78	8.57 ± 1.21	8.72 ± 1.17	<0.001
HbA1c (%)	5.53 ± 1.13	5.36 ± 1.18	9.51 ± 1.01	<0.001
HOMA-IR	2.0 ± 0.83	2.48 ± 0.50	4.40 ± 1.19	<0.05
HOMA-B	104.21 ± 2.08	59.68 ± 6.68	78.91 ± 6.13	<0.05

**Figure 1:**
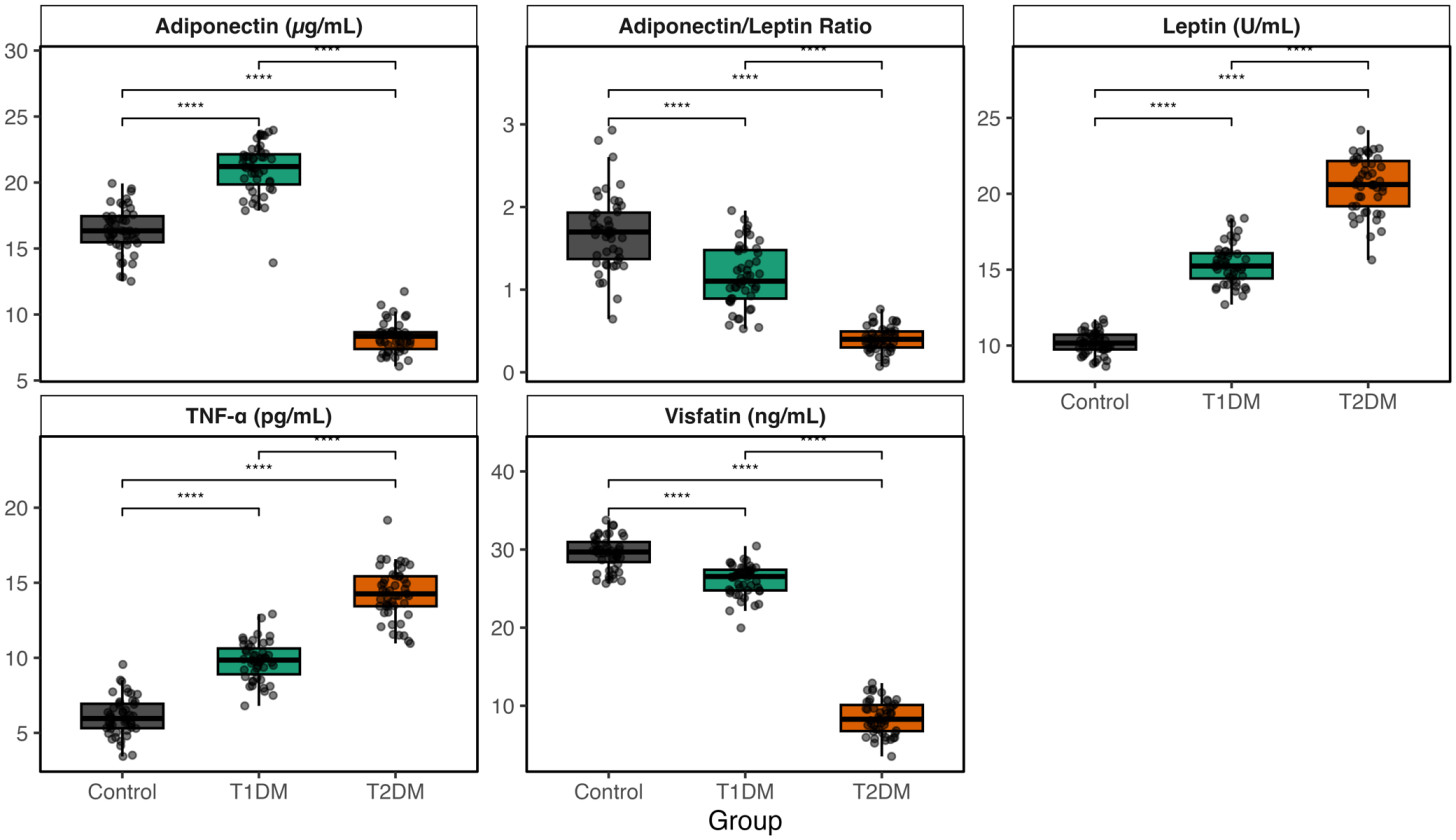
Differential expression of adipokines and inflammatory markers across study groups. Measurements of serum adiponectin (µg/mL), ALR, leptin (ng/mL), TNF-α (pg/mL), and visfatin (ng/mL) were analyzed for Control, T1DM, and T2DM groups. Data for each group are presented as box-and-whisker plots with overlaid individual data points. Each box represents the interquartile range (IQR). Each box is partitioned with a horizontal line at the median, and the whiskers represent the range. The Wilcoxon rank-sum test was used for each between-group comparison. The following signifies the levels of significance: ****p < 0.0001. Adiponectin and ALR levels were significantly diminished in T2DM with respect to Control and T1DM, due to leptin and TNF-α showing evidence of a gradual rise in levels from Control to T2DM. Visfatin levels were highest in the Control group and markedly decreased in the T2DM group, consistent with the differential regulation of inflammatory and metabolic biomarkers across disease states.

Adipokine and inflammatory profiles are presented in **Table 3** and visually illustrated in **Figure 1**. Adiponectin levels were significantly higher in T1DM and markedly reduced in T2DM compared with controls (**Table 3**, p < 0.01; **Figure 1**). Leptin levels showed a progressive increase from control to T2DM (**Table 3**, p < 0.01; **Figure 1**). Consequently, the ALR was significantly reduced in diabetic individuals, particularly in T2DM (**Table 3**, p < 0.01; **Figure 1**).

**Table 3:** Adipokines and inflammatory markers.

Variable	Control (n=47)	T1DM (n=47)	T2DM (n=47)	p-value
Adiponectin (µg/mL)	16.53 ± 1.19	20.68 ± 2.27	8.55 ± 0.50	<0.01
Leptin (ng/mL)	10.19 ± 0.74	15.53 ± 1.08	20.59 ± 1.11	<0.01
ALR	1.68 ± 0.45	1.22 ± 0.30	0.43 ± 0.10	<0.01
TNF-α (pg/mL)	5.87 ± 0.82	10.04 ± 0.88	14.14 ± 0.83	<0.001
Visfatin (ng/mL)	30.00 ± 1.38	26.31 ± 1.08	9.42 ± 1.05	<0.01

TNF-α levels increased stepwise from control to T2DM (**Table 3**, p < 0.001; **Figure 1**), indicating enhanced inflammatory activity. In contrast, visfatin levels were highest in controls and significantly reduced in diabetic groups, with the lowest levels observed in T2DM (**Table 3**, p < 0.01; **Figure 1**). These patterns demonstrate clear group separation across biomarkers.

Correlation analysis revealed that adiponectin and ALR were inversely associated with glycemic parameters and measures of central adiposity, whereas leptin and TNF-α showed positive associations (p < 0.05). Visfatin also demonstrated significant associations with metabolic parameters.

**Figure 2:**
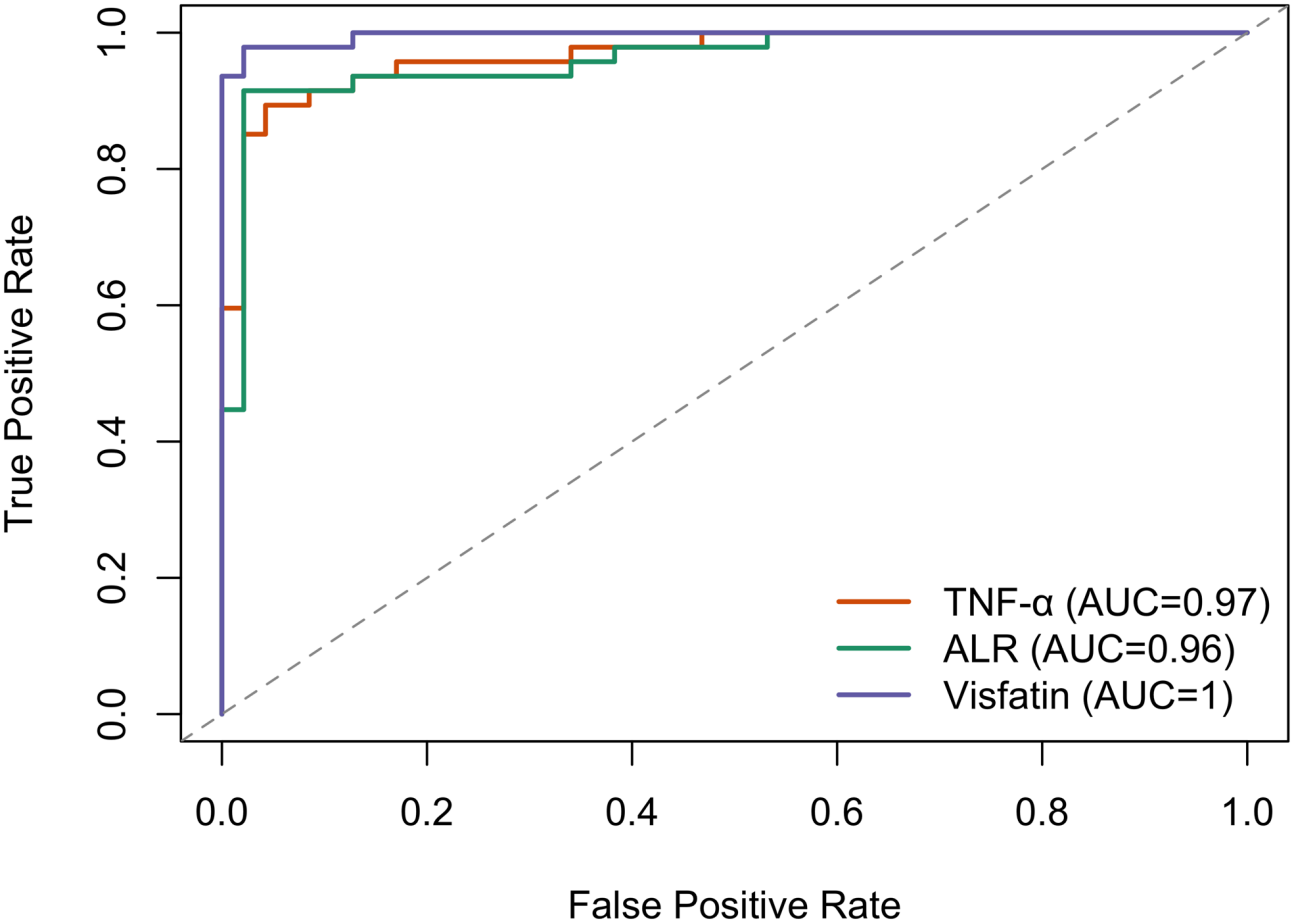
Receiver operating characteristic (ROC) curve analysis of biomarker performance. Receiver operating characteristic (ROC) curves were constructed for TNF-α, visfatin, and ALR to assess how accurately each biomarker measured and differentiated between T2DM patients and controls. Each curve charts sensitivity (true positive rate) and 1 − specificity (false positive rate). The AUC values were TNF-α (AUC = 0.97), ALR (AUC = 0.96), and visfatin (AUC = 0.99). All three markers showed strong discriminatory ability in this exploratory analysis. The dashed diagonal line defines the level of no discrimination (AUC = 0.5).

Multinomial logistic regression analysis identified increased TNF-α levels, reduced ALR, and measures of central adiposity as significant predictors of T2DM. ROC curve analysis demonstrated the strong discriminatory ability of selected biomarkers, as shown in **Figure 2**. TNF-α showed an area under the curve (AUC) of 0.97, ALR an AUC of 0.96, and visfatin an AUC of 0.99, indicating strong performance in distinguishing T2DM from control subjects within this cohort.

## Discussion

This study highlights distinct profiles of adipokines and inflammation, with significant differences in inflammatory and metabolic dysregulation between T2DM and T1DM. These findings are presented through visual (**Figure 1**) and numerical (**Table 3**) data, confirming the value of assessing both adipokines to differentiate diabetes types. The T2DM participants demonstrated a greater degree of central adiposity and greater insulin resistance (**Tables 1**, **2**). This is in line with the established pathophysiology of obesity, metabolic syndrome, and a chronic low-grade inflammatory pathway. In contrast, the T1DM participants tended to be younger and had lower insulin levels (**Table 2**), which would be suggestive of autoimmune destruction of pancreatic beta cells and an insulin-deficient state (13).

The current cohort has participants with T2DM who predominantly display an obesity-related phenotype, with a greater BMI and central fat distribution compared to the other groups. This reflects an obesity-associated phenotype within the T2DM cohort, and the findings may therefore apply mainly to obese T2DM patients and may not extend to non-obese T2DM populations with different adipokine patterns. Other studies that stratified participants by the presence or absence of obesity and diabetes provided a clearer assessment of the separate and combined effects of adiposity and metabolic dysfunction (14, 15). Since this study predominantly included T2DM patients with obesity and did not include non-obese T2DM or obese non-diabetic comparison groups, the independent contributions of obesity and diabetes could not be fully distinguished. Even though decreased levels of adiponectin are linked to obesity and insulin resistance, prior research has indicated that decreased levels of adiponectin are present even in non-obese T2DM patients. Hence, the reduction noted in this study is probably a result of both the adiposity-related and the adiposity-independent metabolic dysfunction.

Leptin levels were higher in the T2DM group compared to controls and T1DM, likely reflecting increased adiposity and leptin resistance (16). In contrast, reduced adiponectin levels indicate impaired insulin sensitivity and metabolic dysfunction (17). The combined effect of elevated leptin and decreased adiponectin contributes to the markedly lower ALR observed in T2DM, reflecting a more pronounced pro-inflammatory and metabolically dysregulated state compared to T1DM (6, 18). Increased levels of TNF-α were observed in both diabetes cohorts, but the most significant levels observed were in the T2DM group (**Table 3**; **Figure 1**), supporting the role of TNF-α as a biomarker of chronic inflammation and insulin resistance (8). While levels of TNF-α were highest in T2DM, a comparable increase was noted in a T1DM cohort. Therefore, TNF-α levels should be viewed as the extent of metabolic and inflammatory dysregulation and not as a true distinguishing marker for different diabetes types (19).

Visfatin, on the other hand, had a more unique response pattern characterized in diabetic groups by lower levels, and lower levels were again present in the T2DM group (20) (**Table 3**; **Figure 1**). Some studies report increased visfatin levels that accompany the presence of metabolic disorders, and the loss of visfatin is posited to be a consequence of the disorder. This may be explained by a number of factors, such as differences in the population under study, the stage of disease, differences in the assay employed, covariates that interact with metabolic visfatin, and the presence of the metabolic disorder (7, 21). The visfatin levels observed in this study may reflect an underlying metabolic process, and further studies are warranted. TNF-α, ALR, and visfatin showed strong discriminatory performance within this cohort in the ROC analysis (22) (**Figure 2**). These ROC results should be regarded as exploratory because no external validation in an independent cohort was performed. The discriminatory performance is therefore likely optimistic and may overestimate the true accuracy of these biomarkers.

## Limitations

This study has several limitations. First, the cross-sectional design limits causal inference. Second, the relatively small sample size, which was not based on a formal power calculation, may reduce statistical power and generalizability. Third, the study included both pediatric and adult subjects with ages ranging from 5 to 70 years, and the groups were not age-matched. Metabolic and inflammatory biomarkers are stage-dependent and also change at different ages. For this reason, age imbalance in group studies is an important confounder. The age imbalance between the T1DM and T2DM groups may also have contributed to the observed differences in biomarkers between the groups. Future studies should consider age-matched groups, pediatric and adult subgroup analyses, or incorporate age into a multivariable modeling framework to disentangle age-related variation in diabetes from other group differences. Fourth, inconsistent medication management across participants may have resulted in treatment-related effects on adipokines and cytokines. Fifth, most participants in the T2DM cohort exhibited an obesity-associated phenotype, making it difficult to apply these findings to T2DM without obesity. Sixth, T2DM participants could only have recently been diagnosed, whereas there were no restrictions on the duration of T1DM. This could have resulted in greater variability in the T1DM participants, which future studies should address by collecting comprehensive duration data. Finally, since the study was performed at a single center, the results should be validated through larger, multicenter research.

## Conclusion

The ALR, visfatin, and TNF-α exhibit distinct patterns across T1DM and T2DM, reflecting differences in metabolic and inflammatory dysregulation. The more pronounced alterations observed in T2DM suggest a greater disruption of adipokine balance and inflammatory activity. These findings support the potential value of integrated adipokine assessment in diabetes characterization; however, they should be considered exploratory and require validation in larger, age- and adiposity-matched cohorts with longitudinal follow-up.

## Data Availability

The original contributions presented in the study are included in the article/supplementary material. Further inquiries can be directed to the corresponding authors.

## References

[1] American Diabetes Association . Diagnosis and classification of diabetes mellitus. Diabetes Care. (2013) 36:S67–74. doi: 10.2337/diacare.29.s1.06.s43 PMC3537273 23264425

[2] Krause M De Vito G . Type 1 and type 2 diabetes mellitus: Commonalities, differences and the importance of exercise and nutrition. Nutrients. (2023) 15:4279. doi: 10.3390/nu15194279 37836562 PMC10574155

[3] Abdalla MMI . Advancing diabetes management: Exploring pancreatic beta-cell restoration’s potential and challenges. World J Gastroenterol. (2024) 30:4339–53. doi: 10.3748/wjg.v30.i40.4339 39494103 PMC11525866

[4] Bin Saleh FS Alibrahim AR Alshahrani AS Aldughaither A Mahzari MM Alharbi WS . Lipid metabolism in type 1 and type 2 diabetes: A comparative cross-sectional study. Med (Baltimore). (2025) 104:e46758. doi: 10.1097/md.0000000000046758 41465900 PMC12747025

[5] Burhans MS Hagman DK Kuzma JN Schmidt KA Kratz M . Contribution of adipose tissue inflammation to the development of type 2 diabetes mellitus. Compr Physiol. (2018) 9:1–58. doi: 10.1002/j.2040-4603.2019.tb00055.x 30549014 PMC6557583

[6] Frühbeck G Catalán V Rodríguez A Gómez-Ambrosi J . Adiponectin-leptin ratio: A promising index to estimate adipose tissue dysfunction. Relation with obesity-associated cardiometabolic risk. Adipocyte. (2018) 7:57–62. doi: 10.1080/21623945.2017.1402151 29205099 PMC5915018

[7] Abdalla MMI . Role of visfatin in obesity-induced insulin resistance. World J Clin cases. (2022) 10:10840–51. doi: 10.12998/wjcc.v10.i30.10840 36338223 PMC9631142

[8] Alzamil H . Elevated serum TNF-α is related to obesity in type 2 diabetes mellitus and is associated with glycemic control and insulin resistance. J Obes. (2020) 2020:5076858. doi: 10.1155/2020/5076858 32089876 PMC7013317

[9] Zadgaonkar U . The interplay between adipokines and body composition in obesity and metabolic diseases. Cureus. (2025) 17:e78050. doi: 10.7759/cureus.78050 40013194 PMC11863173

[10] Chan JCN Malik V Jia W Kadowaki T Yajnik CS Yoon KH . Diabetes in Asia: Epidemiology, risk factors, and pathophysiology. JAMA. (2009) 301:2129–40. doi: 10.1001/jama.2009.726 19470990

[11] Wang L Peng W Zhao Z Zhang M Shi Z Song Z . Prevalence and treatment of diabetes in China, 2013-2018. JAMA. (2021) 326:2498–506. doi: 10.1001/jama.2021.22208 34962526 PMC8715349

[12] Ma RCW Chan JCN . Type 2 diabetes in East Asians: Similarities and differences with populations in Europe and the United States. Ann N Y Acad Sci. (2013) 1281:64–91. doi: 10.1111/nyas.12098 23551121 PMC3708105

[13] Yadav U Kumar N Sarvottam K . Role of obesity related inflammation in pathogenesis of peripheral artery disease in patients of type 2 diabetes mellitus. J Diabetes Metab Disord. (2023) 22:175–88. doi: 10.1007/s40200-023-01221-5 37255816 PMC10225462

[14] Ahmad MS Minaee N Serrano-Contreras JI Kaluarachchi M Shen EYL Boulange C . Exploring the interactions between obesity and diabetes: Implications for understanding metabolic dysregulation in a Saudi Arabian adult population. J Proteome Res. (2024) 23:809–21. doi: 10.1021/acs.jproteome.3c00717 38230637 PMC10846529

[15] Kim H Park S Park J Son Y Kim S Yim Y . National trends in type 2 diabetes mellitus stratified by central adiposity using waist-to-height ratio in South Korea, 2005-2022. Sci Rep. (2024) 14:24273. doi: 10.1038/s41598-024-75002-2 39414865 PMC11484852

[16] Reinehr T Woelfle J Wiegand S Karges B Meissner T Nagl K . Leptin but not adiponectin is related to type 2 diabetes mellitus in obese adolescents. Pediatr Diabetes. (2016) 17:281–8. doi: 10.1111/pedi.12276 25882767

[17] Achari AE Jain SK . Adiponectin, a therapeutic target for obesity, diabetes, and endothelial dysfunction. Int J Mol Sci. (2017) 18:1321. doi: 10.3390/ijms18061321 28635626 PMC5486142

[18] Maahs DM Hamman RF D'Agostino RB Jr Dolan LM Imperatore G Lawrence JM . The association between adiponectin/leptin ratio and diabetes type: the SEARCH for Diabetes in Youth Study. J Pediatr. (2009) 155:133–5. doi: 10.1016/j.jpeds.2008.12.048 19559298 PMC2743881

[19] Chen YL Qiao YC Xu Y Ling W Pan YH Huang YC . Serum TNF-α concentrations in type 2 diabetes mellitus patients and diabetic nephropathy patients: A systematic review and meta-analysis. Immunol Lett. (2017) 186:52–8. doi: 10.1016/j.imlet.2017.04.003 28414180

[20] Yilmaz DT Gul MA Capraz M Demir HD Tekcan A . Investigation of serum visfatin and chemerin levels in type 2 diabetes and obesity patients: Their potential role as clinical and biomarkers. Biomedicines. (2025) 13:2619. doi: 10.3390/biomedicines13112619 41301714 PMC12650185

[21] Dakroub A Nasser SA Younis N Bhagani H Al-Dhaheri Y Pintus G . Visfatin: A possible role in cardiovasculo-metabolic disorders. Cells. (2020) 9:2444. doi: 10.3390/cells9112444 33182523 PMC7696687

[22] Zyśk B Smarkusz-Zarzecka J Cwalina U Gornowicz A Orywal K Bielawska A . Analysis of visfatin concentration and other potential biomarkers associated with MASLD development in saliva and serum of patients with obesity-a pilot study. Nutrients. (2026) 18:652. doi: 10.3390/nu18040652 41754169 PMC12943479

